# Bacterial Loads on Skin of Unclipped Gluteal Sites Following Treatment with 70% Isopropyl Alcohol-Soaked Swabs in Dairy Cows

**DOI:** 10.3390/vetsci2030206

**Published:** 2015-08-14

**Authors:** Peter D. Cockcroft, Helen E. Redfern

**Affiliations:** 1 School of Animal and Veterinary Sciences, University of Adelaide, Adelaide, SA 5371, Australia; 2 Glenthorne Veterinary Centre, Uttoxeter, Staffordshire ST148EB, UK; E-Mail: stuart@glenthornevets.co.uk

**Keywords:** bacteria, injection, skin, isopropyl alcohol, cattle

## Abstract

The aim of this study was to compare the bacterial load of unclipped gluteal skin in dairy cows following either no treatment or treatment with a standard 70% isopropyl alcohol-based skin treatment protocol. Twenty Holstein-Friesian dairy cows from a commercial dairy herd in Cambridgeshire, England, were used in this randomised, blinded, controlled study. On each of the experimental cows an area of unclipped gluteal skin on one side of the pelvis was treated with swabs soaked in 70% isopropyl alcohol-based using a standard protocol and a contra-lateral area of skin was left untreated as a control. All the experimental skin sites were sampled using a swab followed by bacterial culture and quantitative analysis of bacterial load. There was a statistically significant decrease in the bacterial colony forming units per mL for the isopropyl-alcohol treatment group when compared to the control group (*p* ≤ 0.01). There was a 58% reduction in the median bacterial load of the treated sites when compared to the bacterial load of the untreated sites. This study has demonstrated that the treatment protocol will reduce the skin bacterial load.

## 1. Introduction 

Intra-muscular injections in cattle are a common procedure performed by veterinarians in cattle practice. There is a wide variety of bacterial flora present on cattle skin including *Bacillus spp.* and non-haemolytic *Staphylococcus spp.* which may lead to abscess formation [1]. Intra-muscular injections of non-antimicrobial substances such as hormonal treatments are common in dairy cattle practice. The gluteal muscles or the neck muscles are the most common injection sites with the latter being preferable in beef animals to minimise the economic losses of muscle damage. Abattoir surveys in the USA beef industry have reported injection lesions incidences of 11.4% in 1995 decreasing to 2.1% in 2000 in top sirloin butts from fed steer and heifers [2]. A further review has further highlighted the economic importance of injection site lesions damage in beef cattle [3]. The incidence of abscesses or infection following injections in cattle is unknown.

There are no published surveys regarding skin preparation protocols used by veterinarians prior to intra-muscular or subcutaneous injections in cattle or their frequency of use. Ten published descriptions of intra-muscular injection procedures for cattle identified by the first author had no reference to skin preparation prior to injection. In the experience of the first author skin preparation is rarely performed in cattle practice prior to intra-muscular injections. If anti-bacterial skin treatment is performed cotton wool swabs soaked in 70% isopropyl alcohol are most frequently used. The aim of this study was to compare the bacterial load of unclipped gluteal skin in dairy cows following either no treatment or treatment with a 70% isopropyl alcohol-based skin treatment protocol.

## 2. Experimental Section 

Twenty Holstein-Friesian dairy cows from a commercial UK dairy herd located in Cambridgeshire, England were used in the study. The animals were at grass at the time of the study. On each cow two experimental unclipped areas of pelvic gluteal skin on either side of the pelvic were marked by a circular chalk mark 3 cm in diameter. These two experimental areas of gluteal skin on each cow were randomly allocated to the treatment and control groups. The 20 areas of skin allocated to the treatment group were prepared using a standard protocol which consisted of rubbing the skin with a swab soaked to saturation in 70% isopropyl alcohol. The swab was rotated 10 times over the marked area. One minute after treatment the skin was sampled by an operator blinded to the treatments. A sterile swab soaked in sterile distilled water was placed upon the skin and rotated three times. The swab was then placed in charcoal transport medium and submitted for bacteriological analysis within 2 hours of the sampling procedure. This sampling procedure was repeated on the contralateral marked pelvic area of untreated gluteal skin. 

At the laboratory each swab was vortexed in 1 mL of sterile saline for 1 minute. The supernatant was then serially diluted using sterile saline for quantitative bacterial analysis. The effective dilutions were 1: 00, 1:1000, 1:10,000 and 1:100,000. Aliquots (100 µL) of each dilution were then pipetted onto the centre of blood agar plates and spread using a sterile L-shaped plastic rod until the surface was dry. All plate treatment was carried out in biological safety cabinet with a 30-minute plate drying time pre and post inoculation. Blood agar plates were incubated at 36.9 °C in an aerobic environment for 24 hours. Plates were read using a plate counter to ascertain the number of CFUs. Bacterial growths were quantified as colony forming units (CFUs) per mL of sample. The mean of all dilutions with colonies present was used as the sample value. 

## 3. Results 

Table 1 presents the descriptive statistics of the study. There was a statistically significant decrease in the CFUs per mL for the isopropyl-alcohol treatment group when compared to the control group (*p* ≤ 0.01, Mann-Whitney U-test (SPSS, IBM)). The median bacterial load of the treated sites was 42% of bacterial load of the untreated sites. There was no statistically significant difference between either the right and left treatment sites (*p* > 0.05, Mann-Whitney U-test (SPSS, IBM)) or the right and left control sites (*p* > 0.05, Mann-Whitney (SPSS, IBM)).

**Table 1:** Bacteriological culture results for the isopropyl alcohol treatment group and the control group of gluteal skin injection sites.

Parameter	Isopropyl Alcohol Treatment Group	Control Group
Number of cows	20	20
Median (CFUs/mL)	6950	16,567
Range (CFUs/mL)	1300–21,650	400–96,500
Percentiles 25th–75th (CFUs/mL)	2,225–11,200	12,979–31,350

## 4. Discussion 

There is currently no evidence-based accepted protocol for the treatment of skin before an intra-muscular or sub-cutaneous injection is administered in cattle. Abscesses deep in the gluteal region are reported in abattoirs in the UK although this data is unpublished [4]. It is uncertain what, if any reduction in bacterial skin load including endospores is necessary prior to injection to preclude abscess formation or the possible potentiation of blackleg *(Clostridium chauvoie*) in cattle as described in the horse [5]. 

In human medicine it is considered best practice to perform intra-muscular injections without swabbing if the skin at the injection site is clean [6]. In equids it is advised that every single injection should be performed as antiseptically as possible and disinfecting or cleaning the injection site must be carried out before each injection [7]. In equids the clipping of the hair at the injection site is only considered necessary if the hair is long [8] and shaving the hair is not an option because it produces more reactions of the skin, which leads to a greater risk of infection [9].

This study has demonstrated that a treatment protocol with swabs soaked in 70% isopropyl alcohol will reduce the skin bacterial load but not eliminate all bacteria. The effect may be due to either the physical removal of bacteria by rubbing with a swab or the action of the 70% isopropyl alcohol or a combination of both. Further work is required to evaluate the impact of skin treatment in relation to the bacterial load introduced by the hypodermic needle and the consequences of the introduction. 

## 5. Conclusions

This study has demonstrated that a treatment protocol with swabs soaked in 70% isopropyl alcohol will reduce the skin bacterial load, but not eliminate all bacteria.
